# DNA Fingerprinting Validates Seed Dispersal Curves from Observational Studies in the Neotropical Legume *Parkia*

**DOI:** 10.1371/journal.pone.0035480

**Published:** 2012-04-13

**Authors:** Eckhard W. Heymann, Kathrin Lüttmann, Inga M. Michalczyk, Pedro Pablo Pinedo Saboya, Birgit Ziegenhagen, Ronald Bialozyt

**Affiliations:** 1 Abteilung Verhaltensökologie & Soziobiologie, Deutsches Primatenzentrum, Göttingen, Germany; 2 AG Naturschutzbiologie, Philipps-Universität Marburg, Marburg, Germany; 3 Mobile DNAnalyse, Ebsdorfergrund, Germany; 4 Facultad de Ciencias Forestales, Universidad Nacional de la Amazonía Peruana, Iquitos, Peru; University of Guelph, Canada

## Abstract

**Background:**

Determining the distances over which seeds are dispersed is a crucial component for examining spatial patterns of seed dispersal and their consequences for plant reproductive success and population structure. However, following the fate of individual seeds after removal from the source tree till deposition at a distant place is generally extremely difficult. Here we provide a comparison of observationally and genetically determined seed dispersal distances and dispersal curves in a Neotropical animal-plant system.

**Methodology/Principal Findings:**

In a field study on the dispersal of seeds of three *Parkia* (Fabaceae) species by two Neotropical primate species, *Saguinus fuscicollis* and *Saguinus mystax*, in Peruvian Amazonia, we observationally determined dispersal distances. These dispersal distances were then validated through DNA fingerprinting, by matching DNA from the maternally derived seed coat to DNA from potential source trees. We found that dispersal distances are strongly right-skewed, and that distributions obtained through observational and genetic methods and fitted distributions do not differ significantly from each other.

**Conclusions/Significance:**

Our study showed that seed dispersal distances can be reliably estimated through observational methods when a strict criterion for inclusion of seeds is observed. Furthermore, dispersal distances produced by the two primate species indicated that these primates fulfil one of the criteria for efficient seed dispersers. Finally, our study demonstrated that DNA extraction methods so far employed for temperate plant species can be successfully used for hard-seeded tropical plants.

## Introduction

Seed dispersal, i.e. the process of displacing seeds away from the maternal plant, creates the template for the next steps within the so-called “seed dispersal loop” [1] like secondary seed dispersal, seed predation, germination and seedling establishment. The spatial pattern of seed dispersal influences plant demography and population structure, and the composition of plant communities [1], [2]. One of the major challenges for the analysis of the spatial pattern of seed dispersal has been the difficulty of following the fate of individual seeds and thus to quantify the contribution of individual plants to seeds found at a given location [3]. For wind-dispersed seeds, inverse modelling has been employed successfully to estimate seed dispersal curves from frequency distributions of seed dispersal distances [2]. For zoochorous seed dispersal, dispersal curves are often modelled from known or estimated gut transit times of seeds in combination with animal movement patterns [4], [5].

A more direct approach to obtain proper estimates of seed dispersal distances is to monitor the behaviour and movement of frugivores directly and continuously and to calculate dispersal distances accordingly. This holds particularly true when the occurrence of seeds in defecations can be matched to feeding bouts by frugivores in a specific plant individual. An unambiguous assignment of these seeds to their origin is therefore possible if the following criterion is fulfilled: between the feeding on fruits of a particular plant species and the defecation of seeds from that species, no other feeding plant of the same species must have been visited. This kind of observation is often possible with habituated primate groups that allow close observation of feeding and defecation [6]. Several studies of primate seed dispersal have calculated seed dispersal distances in this way [7]–[10]. Still, the reliability of these calculations has yet to be validated. Especially long distance dispersal events could be underestimated using such an observational approach. If further feeding trees of the same species are visited in between feeding in the first tree and defecation, the detected seeds can no longer be attributed to a specific tree and therefore are lost for calculating dispersal distances using observational data only. Furthermore, if the seed disperser switches between two (or more) feeding trees in a reasonable time span (gut transit time) additional short distance dispersal should occur. This time short dispersal distances would be missing. A further shortcoming might be the group size of habituated primate groups. Not all members of that group might be in the visibility range and it is possible that one individual does feed on a tree en route. Thus distances might be overestimated.

To overcome these limitations, genetic methods can be used. Especially, DNA fingerprinting is the method of choice for unambiguous seed source identification. This is achieved by comparing the DNA fingerprint of potential mothers with that of the seed coat of dispersed seeds [11], [12]. Since in angiosperms, the seed coat is of pure maternal origin [13] a matching algorithm is sufficient to explicitly assess seed dispersal distances of defecated seeds. This approach is free of any assumptions on gut transit times and animal movement patterns which may introduce a bias into calculations of seed dispersal distances.

In this study, we use both an observational approach and the genetic methods described above to assign seeds recovered from tamarin faeces to source trees. From both procedures we calculate frequency distributions of seed dispersal distances produced by two small (250–600 g), frugivorous Neotropical primate species, the tamarins *Saguinus fuscicollis* and *Saguinus mystax*, living in mixed-species troops in Peruvian lowland Amazonia [14], and fitted dispersal curves to these distributions. We focus on a specific tree genus (*Parkia*, family Fabaceae), which is an important food resource for tamarins [15], [16] and for which the tamarins are most likely the only seed dispersers in the study area, as determined by focal tree observations (unpubl. data). Where the criterion quoted above can be applied, behaviourally determined dispersal distances and dispersal curves, should be concordant with the genetically determined dispersal distances and curves. If this could be shown, estimates of dispersal distances from our earlier field work where genetic methods were not yet available and from other studies can be considered as reliable and provide valuable data sets for further long-term and comparative spatial analyses of seed dispersal processes in (Neotropical) plant-disperser systems.

## Results

### Individual identification and genotype matching

During seed collection in the field we could assign 129 of the 133 collected seeds to 12 different mother trees according to the rigid criterion for observational data (Table 1). The combination of all seven marker loci was powerful enough for individual identification (*PI* = 2.6×10^−12^). Out of 133 seed coats, 102 could exactly be matched to 12 different mother trees (Table 1). When one mismatch was allowed, another 21 seed coats could be matched to the same 12 mother trees. The remaining 10 seed coats mismatched at more than one locus with all of the potential adult trees and were not considered for calculating dispersal distances. Overall, we observed 19 seeds where the assignment to the mother tree from observational data did not match the one from genetic analysis.

**Table 1:** Number of seeds assigned to source trees and mean dispersal distances per source tree.

Source tree	# seeds assigned to source tree		Mean dispersal distance [m]	
	Observational	genetic	observational	genetic
005	-	1	-	163
015	37	25	142	156
018	12	20	199	185
020	3	-	198	-
025	5	4	273	225
042	2	2	162	162
046	6	5	276	223
049	1	-	350	-
052	14	12	159	163
055	30	34	149	150
056	-	1	-	337
057	3	3	404	404
058	15	15	138	165
090	1	1	413	413
Total	129	123		
Mean over all source tree ± SD			239±103	229±99

### Seed dispersal distances

**Figure 1:**
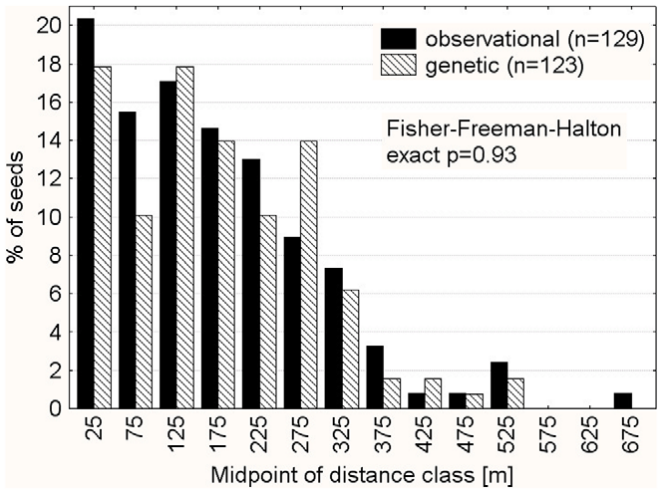
Frequency distribution of dispersal distances as determined by observational and genetic methods. Observational methods used the following criterion for inclusion of seeds: between the feeding on fruits of a particular *Parkia* individual and the defecation of *Parkia* seeds, no other *Parkia* individual must have been visited. Genetic methods used DNA fingerprinting to assign seeds to a source trees.

**Figure 2:**
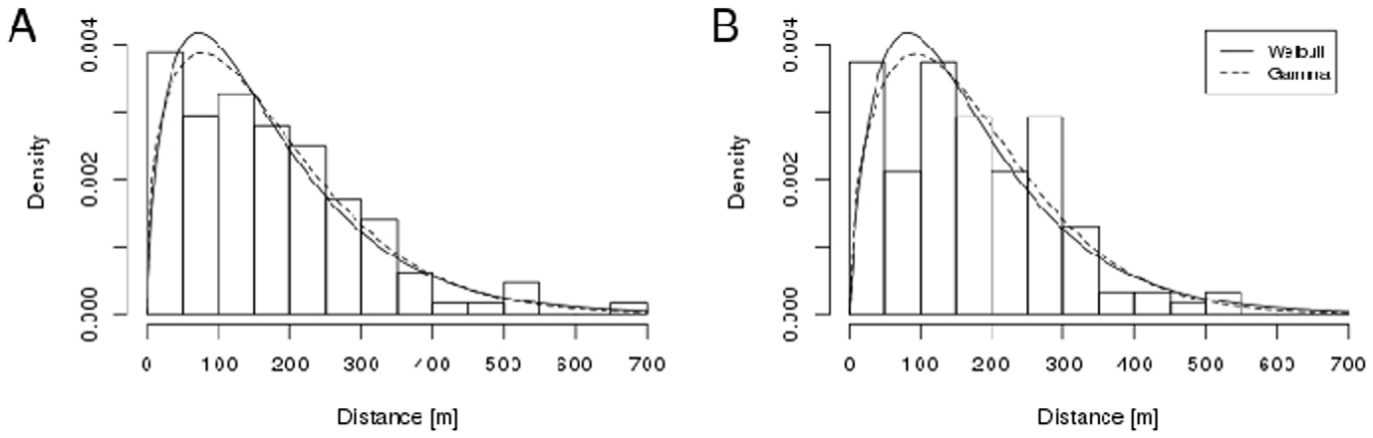
Distribution fitting to the dispersal distance distributions obtained with observational (A) and genetic methods (B). Fitted distributions do not differ between genetically and observationally obtained dispersal distance distributions.

The frequency distributions of dispersal distances generated by the two different methods (observational *vs.* genetic) are highly concordant (Fisher-Freeman-Halton test p = 0.93; Fig. 1). Both distributions are similarly right-skewed (observations: 1.6, genetic: 1.45). Minimum dispersal distances were 9.5 m (observational and genetic), maximum dispersal distances 656 m (observational) and 513 m (genetic). None of the dispersed seeds ended directly beneath the crown of the source tree. Despite the difference between the maximum dispersal distance of both methods, the fitted distributional parameters are very similar (Gamma shape parameter 1.90 *vs.* 1.88; Weibull scale parameter 189.9 *vs.* 193.5; Fig. 2, Table 2) and hint at a good match between both datasets. Mean dispersal distances over all source trees do not differ between observational and genetic methods (randomization test: p = 0.8166; Table 1).

**Table 2:** Shape and scale parameters of distributions fitted to observationally and genetically determined seed dispersal distances.

		Weibull parameter		Gamma parameter	
		scale	shape	shape	rate
Full data set (123 seeds)	observational	189.9	1.39	1.70	0.01
	genetic	193.6	1.51	1.88	0.01
Perfect matches (102 seeds)	observational	208.1	1.48	1.81	0.01
	genetic	200.1	1.56	1.89	0.01

## Discussion

Keeping track of the fate of individual seeds represents a major challenge in seed dispersal studies. This challenge is particularly strong with seeds that pass through the gastro-intestinal track of an animal vector, and thus disappear from observers' view after ingestion. Studies which calculate seed dispersal distances and fitted seed dispersal curves from observational studies make the assumption that seeds voided with faeces can be assigned to a source tree when the outlined rigid criterion (see Introduction) is applied. In our study, we validated for the first time the observational approach through the employment of DNA fingerprinting that allows an unambiguous assignment of seeds to their source tree. We have shown that the seed dispersal distances and fitted dispersal curves resulting from behavioural observations can be considered as reliable, as suggested by the high concordance of observationally and genetically determined dispersal distances and curves, respectively. The application and compliance of a rigid criterion for inclusion of seeds into the observational determination of seed dispersal distances is essential for the reliability of the behavioural approach. Studies that applied this criterion [7]–[10] thus can be considered as yielding valid results. Thus, previous data sets based on observational work alone can be legitimately used in the analyses of spatial patterns of tamarin seed dispersal. Deviations between observationally and genetically determined mean seed dispersal distances per mother tree are generally small (Table 1) and would not affect the general spatial pattern.

Compliance of the rigid criterion may not be possible for each plant-endozoochorous dispersal system, e.g. when the tamarins feed on *Leonia cymosa* (Violaceae) – a species that shows spatial clustering – they visit several trees in succession [17]. In this case, only genetic methods will provide reliable estimates of seed dispersal distances.

The distributions of dispersal distances in our study are strongly right-skewed. This is in accordance with findings from other seed-dispersing vertebrates where dispersal distances were determined either by direct observation or through modelling from movement patterns and gut transit times [18]–[20], or through genetic analyses [21]. While in our study a large proportion of seeds were found in the smallest distance class, none actually landed below the source tree. Estimates based on 1664 dispersal events for 39 plant species dispersed by *S. fuscicollis* and *S. mystax* showed that only 2.5% of seeds landed within 10 m of the feeding plant, i.e. below or close to the crown (<0.6% of plants exploited by tamarins at EBQB have a crown radius >10 m) (Knogge et al. unpublished data). Thus, these two primate species consistently transport seeds away from the area of supposedly high predation risk below the crown of the source tree, which contributes to the efficiency of seed dispersal [22]. Seed coats of *Parkia* are very hard and thus the extraction of DNA provided a technical challenge. The method employed in our study, which is based on previous work by Ziegenhagen et al. [23] proved to be successful. This opens the path for employing this method for many other tropical plant species with hard or very hard seed coats. Hard seed coats are perhaps typical for many tropical plant species [24] – particularly those that provide a soft pulp to attract seed dispersers – as an adaptation against habitual predation by specialized seed predators and accidental predation by seed dispersers. This will strongly expand the options for including genetic methods in seed dispersal studies in the tropics.

Apart from *Parkia*, tamarins disperse the seeds of a wide variety of their food plant species [16], [25]. Tamarins can persist in disturbed areas and secondary forests where populations of large-sized primates area absent or strongly reduced. Therefore, tamarin seed dispersal can become particularly important for vegetation regeneration [25], [26]. Analysing the spatial patterns of tamarin seed dispersal is an essential component for understanding this ecological function. Our genetic validation of observationally determined dispersal distances will contribute to this by allowing the inclusion of previously collected data for a long-term monitoring of tamarin seed dispersal and also the analysis of potential changes of seed dispersal patterns.

## Materials and Methods

### Natural history information on Parkia

The genus *Parkia* belongs to the family Fabaceae, subfamily Mimosoideae, and has a pan-tropical distribution [27], [28]. In the Neotropics, it is represented by 19 or more species [27], [29] that mostly grow as medium to tall or even emergent trees in *terra firme*-forests [27], [30]. The compound inflorescences are pollinated by bats and bees [27], [31]. Seed dispersal is mainly by primates and terrestrial rodents [32]. The following primate species are known seed dispersers of *Parkia*: tamarins (*S. fuscicollis* and *S. mystax*), spider monkeys (*Ateles belzebuth*) and woolly monkeys (*Lagothrix cana* and *Lagothrix poeppigii*) [15], [16], [33], [34]. Bruchid beetles, ants, large parrots, several primate species (*Cebus apella*, *Pithecia albicans*, *Cacajao calvus ucayalii*) and terrestrial ungulates and rodents prey on *Parkia* seeds [16], [32], [35], [36] [own observations]. At our study site, *S. fuscicollis* and *S. mystax* are the only frugivores that have been observed to disperse *Parkia* seeds during focal tree observations.

### Study site and field methods

The study was carried out at the Estación Biológica Quebrada Blanco (EBQB), located at 4°21′S 73°09′W in the Amazon lowlands of north-eastern Peru. The site is characterised by rainforest of the *bosque de altura* (*terra firme*) type [37]. For further details of the site see [38].

A mixed-species troop of four *S. fuscicollis* and six *S. mystax* was observed by KL on 67 days between May and September 2008. Observations started when the tamarins left a sleeping site in the early morning and terminated when they retired to a sleeping site in the afternoon. Data on the location of the troop within the home-range area were recorded every 15 min with a Garmin GPSMap® 76CSx. The start and end time of each visit to a food plant was recorded. Defecations of the tamarins that contained one or more *Parkia* seeds were collected and mapped with GPS. A total of 133 seeds were collected. To avoid mouldering, the seeds were stored in a saturated NaCl-solution until DNA extraction.

In order to genetically match the seeds to their source tree after genotyping, leaves were sampled from all 99 *Parkia* trees (height≥1.3 m, DBH≥20 cm) within the home-range area of the study group. All these *Parkia* trees were mapped with GPS. Leaves were dried and stored together with silica gel in plastic bags until DNA extraction.

### DNA extraction and DNA fingerprinting of seed coats and leaves

Since the woody seed coats of *Parkia* trees are extremely hard, it was not possible to directly open and separate them from other seed tissue. Therefore, seeds were incubated in distilled water at room temperature until they burst open by themselves or until a mechanical opening with pincers was possible (approx. after 1 week of incubation). Afterwards, we dissected seed coats from all other tissues. To extract total genomic DNA from seed coats and leave samples, 100 mg of each sample were homogenised with the help of a Retsch shaking mill (Retsch, Hilden, Germany) following the protocol of [23]. DNA extraction followed an ATMAB-based mini-preparation protocol [39] with an additional and final treatment with 0.5 µl RNase at 37°C for 30 min. Concentration of genomic DNA was measured using a UV-Photometer (GeneRay, Biometra Göttingen, Germany).

**Figure 3:**
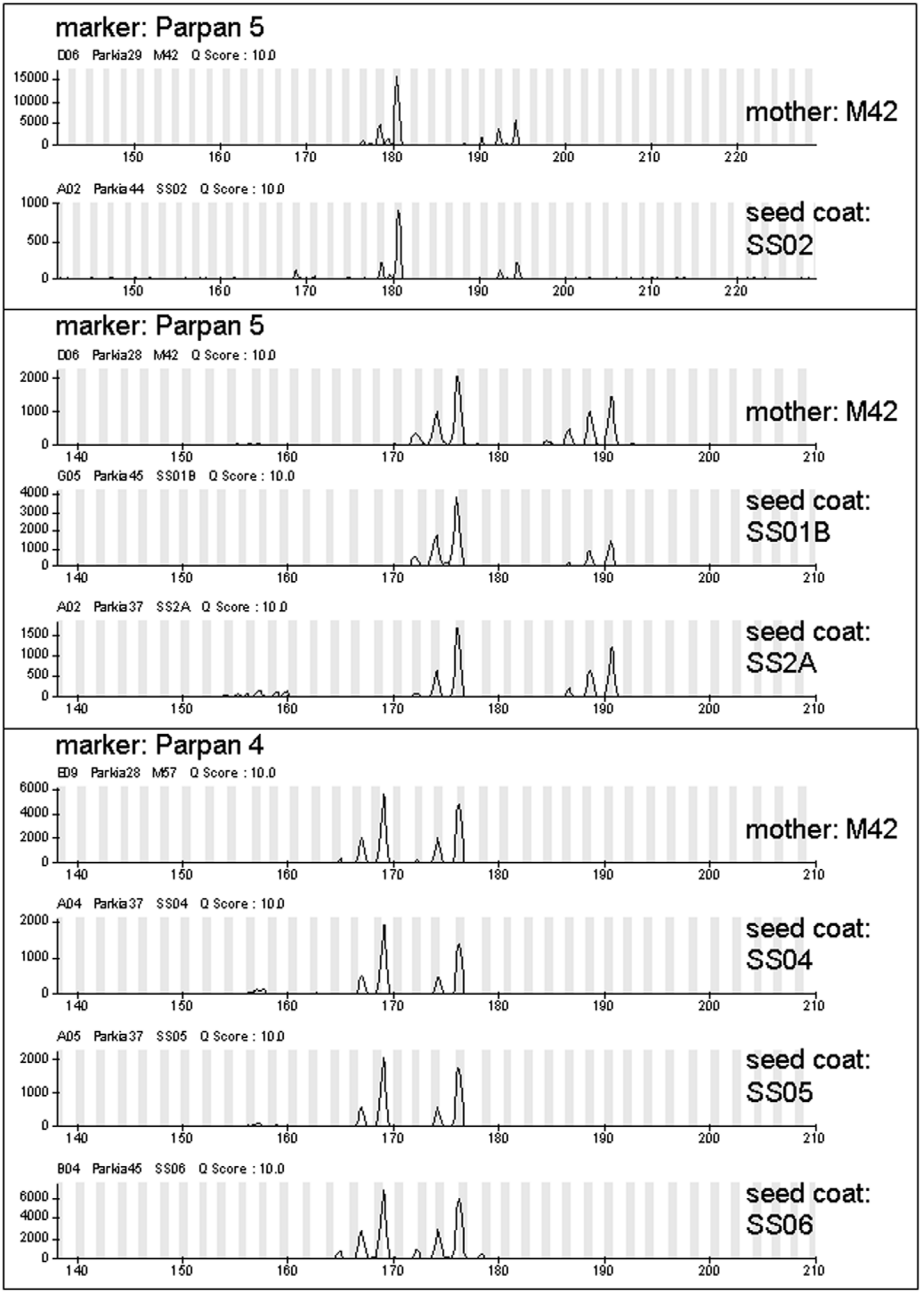
Examples of electrofluorograms showing the comparison between tissue from the mother tree and from seed coats for markers Parpan 4 and Parpan 5. x-axis: fragment length (number of bases), y-axis: signal intensity. In all cases the diploid heterozygous genotypes exhibit more than just two peaks. This is due to stuttering which is common. The true allele is the one with the highest signal intensity.

For DNA fingerprinting all seed coat and leaf samples were analysed at seven highly polymorphic nuclear microsatellite (nSSR) loci, previously characterised in *Parkia panurensis*: Parpan3, Parpan4, Parpan5, Parpan9, Parpan14, Parpan15, Parpan21 [40]. PCR amplifications were carried out in a Thermocycler (Biometra, Göttingen, Germany) using fluorescent labelled primers. PCR protocols are specified in [40]. Numbers of cycles were increased for the seed coats following the procedure with other exocarp material [41]. Amplification products were separated by capillary electrophoresis using MegaBACE 1000 (GE Healthcare, Uppsala, Sweden) automatic sequencer. Alleles were sized using the size standard MegaBACE ET400-R (GE Healthcare) and the software MegaBACE Genetic Profiler 2.2 (GE Healthcare). An example of electrofluorograms comparing genotypes of trees and seeds is shown in Fig. 3. The software Micro-Checker 2.2.3 [42] was used to test for unexpected allele sizes and missing data. Thus, the resulting non-fitting allele sizes were corrected manually afterwards. The probability of identity (*PI*; [43]) was calculated for the potential mother trees using the computer programme GenAlEx version 6.4 [44]. This value describes the probability of two individuals randomly sharing the same genotype in the data set. Therefore, it is a measure for the statistical power of individual identification with the chosen marker combination. None of the 99 *Parkia* trees within the study area shared the same genotype with respect to the seven analysed loci.

### Attribution of seeds to source trees

Four species of *Parkia* are found at our study site: *P. panurensis*, *P. igneiflora*, *P. multijuga* and *P. nitida*. While these species can be separated morphologically, genetic analyses with Structure 2.3.1 [45] of 456 individuals (adult trees, saplings, seedlings, and seeds) revealed only two clusters, one conformed of individuals identified as *P. panurensis*, the other conformed by the other three species that could not clearly be separated genetically [unpublished results]. Three species – *P. panurensis*, *P. igneiflora*, *P. multijuga* – have their seeds dispersed by tamarins at our site.

Seeds were attributed to their source trees by comparing the multilocus genotypes of leaves and seed coats, using the “Multilocus” option “Matches” of the Software GenAlEx version 6.4 [44]. Missing data were ignored when searching matches. Multilocus genotypes of seed coats and adult trees, which matched at all analysed nSSR loci were considered to be identical and therefore a mother-offspring pair. Additionally, one mismatch among multilocus genotypes was allowed, if the following conditions were met: The mismatch occurred in the shape of a homozygote genotype of the seed coat of interest while the putative mother tree exhibited a heterozygote genotype containing the allele of the seed coat and *vice versa*. Such mismatches were attributed to allelic dropout, which is a common phenomenon with both low-quality and high-quality DNA [46], [47] and which was observed for DNA-extracts from seed coats and leaves. However, just one dropout was tolerated per multilocus genotype in order to preserve most of the discrimination power of the marker combination.

### Calculation of dispersal distances and fitting dispersal curves

Dispersal distances were calculated as the linear distance between (a) a *Parkia* tree where the tamarins were observed feeding and the site of subsequent defecation; and (b) the genetically identified source tree and the site of defecation. The two resulting frequency distributions of dispersal distances were compared with the Fisher-Freeman-Halton test using the function “fisher” of the R-package “stats” version 2.11.1 [48]. The α-level was set at 0.05. Skewness and kurtosis of the distribution was calculated using the R-package “moments” version 0.12 [49].

The dispersal curves were fitted to the two datasets of dispersal distances using the function “fitdistr” of the R-package “MASS” [50]. This way of fitting distribution functions is completely free of the chosen distance classes but fits the chosen distribution to individual dispersal events. The comparison of the fitted parameters between both datasets is expected to provide a good estimate of the match. Mean dispersal distances over all source tree were compared with a randomization test (10000 permutations) in SsS 2.0e.
